# Independent reliability and availability analyses of modified classification for migrated lumbar disc herniation

**DOI:** 10.1186/s13018-023-03688-7

**Published:** 2023-03-14

**Authors:** Wenshuo Gao, Wei Zhang, Hao Pan, Dong Wang

**Affiliations:** Department of Orthopaedics, Hangzhou TCM Hospital Affiliated to Zhejiang Chinese Medical University (Hangzhou Hospital of Traditional Chinese Medicine), No. 453 Tiyuchang Road, Xihu District, Hangzhou, 310000 Zhejiang Province People’s Republic of China

**Keywords:** Classification, Migrated lumbar disc herniation, Reliability

## Abstract

**Study design:**

Retrospective cohort study.

**Objective:**

The purpose of this study was to evaluate the reliability of modified classification system of migrated nucleus pulposus and its clinical application value.

**Methods:**

We retrieved 1000 lumbar MRI of different patients in Hangzhou Hospital of Traditional Chinese Medicine from January 2016 to December 2019 for interpretation, and screened 105 migrated lumbar MRI for inclusion in the study. Three spinal surgeons made classification according to the modified classification method. Two weeks later, the sorting data of the patients were shuffled and the classification was judged by three doctors again. The consistency and repeatability of the improved classification were evaluated by Kappa coefficient. The general data of the included patients were collected. The patients were followed up for 2 years, and the risk factors of surgical treatment of patients with migrated lumbar disc herniation were analyzed. The treatment plan, surgical approach, operation time, VAS score, ODI score and other relevant data of the included patients were collected to evaluate the guiding effect of the classification system on clinical practice.

**Results:**

In this study, the incidence of migrated lumbar disc herniation was about 10.5%, and most of the patients were male. Patients with higher BMI are more likely to develop this disease. Our study confirmed that the modified classification has moderate to high confidence. During the 2-year follow-up period, 66 patients (62.9%) were treated conservatively, and the patients with conservative treatment were mainly A2 and B2 type (59.1%). Thirty-nine patients (37.1%) underwent surgical treatment. The patients recovered well after operation, and the low back pain and ODI index were significantly improved at 1 year after operation (*P* < 0.05). We suggest that type A1 and B1 migrated nucleus pulposus can be removed by posterior approach. For type A2, B2, C1, C2, the lateral approach is recommended to remove the nucleus pulposus directly. Logistic regression and ROC analysis showed that disease duration (≥ 1 year) and BMI (≥ 24) maybe were risk factors for surgical treatment of patients with migrated lumbar disc herniation.

**Conclusion:**

The modified classification has good reliability. In the current study, the experience level of spine surgeons does not affect the reliability of the classification system. Our study confirmed that this classification has a good reference value for guiding the treatment plan and the choice of surgical approach.

Migrated Lumbar Disc Herniation (MLDH) is a special type of Lumbar Disc Herniation in which the nucleus pulposus fragment prolapsed migrated from the spinal canal, which is common in clinical practice [1]. Previous literature reported that the clinical incidence of MLDH is 1.7–3% [2, 3]. Migrated nucleus pulposus tissue can not only cause physical compression to the nerve root, but also cause aseptic inflammatory response at the same time, resulting in severe congestion and edema of the affected nerve root, which can lead to severe neurological dysfunction in the long term [4–6]. Therefore, clinicians should pay great attention to the diagnosis and treatment of MLDH.

Imaging localization and classification are very important for prognosis determination and surgical planning [7]. For migrated discs, there is a correlation between different locations of the nucleus pulposus and clinical symptoms. For example, the migrated nucleus pulposus, located in the foramen area, often leads to severe numbness and radiation pain in the lower limbs. For patients with MLDH, lumbar MRI can clearly show the specific location and shape of the migrated nucleus pulposus, and the images obtained are more complete and comprehensive, which is helpful to guide clinical treatment [8, 9]. At present, there are many MRI-based image classification methods, and two are recognized by most scholars. One is the transection site classification method proposed by Hu et al. [10]. The other is the sagittal classification method proposed by Lee et al. [11]. However, in clinical application, neither of these two types alone can show the location of prolapse migrated nucleus pulposus stereoscopically. Referring to these two types of classification, our team recently proposed a modified classification of migrated nucleus pulposus. On the basis of ignoring the primary segment, the two types were fused and simplified into type A, B, C and two groups of subtypes of upward and downward mobilization according to the degree of mobilization of nucleus pulposus in the transverse and sagittal planes (Table 1 and Fig. 1). The purpose of this study is to verify the reliability of the modified classification system of migrated nucleus pulposus, and to evaluate its clinical application and guiding the surgical approach of percutaneous transforaminal endoscopic discectomy (Table 2).

**Table 1:** Modified classification of migrated nucleus pulposus

Sagittal zoning	Direction	Transverse zoning		
		Zone 1	Zone 2	Zone 3
I	Upward	A1	B1	C1
II				
III	Downward	A2	B2	C2
IV

**Fig. 1:**
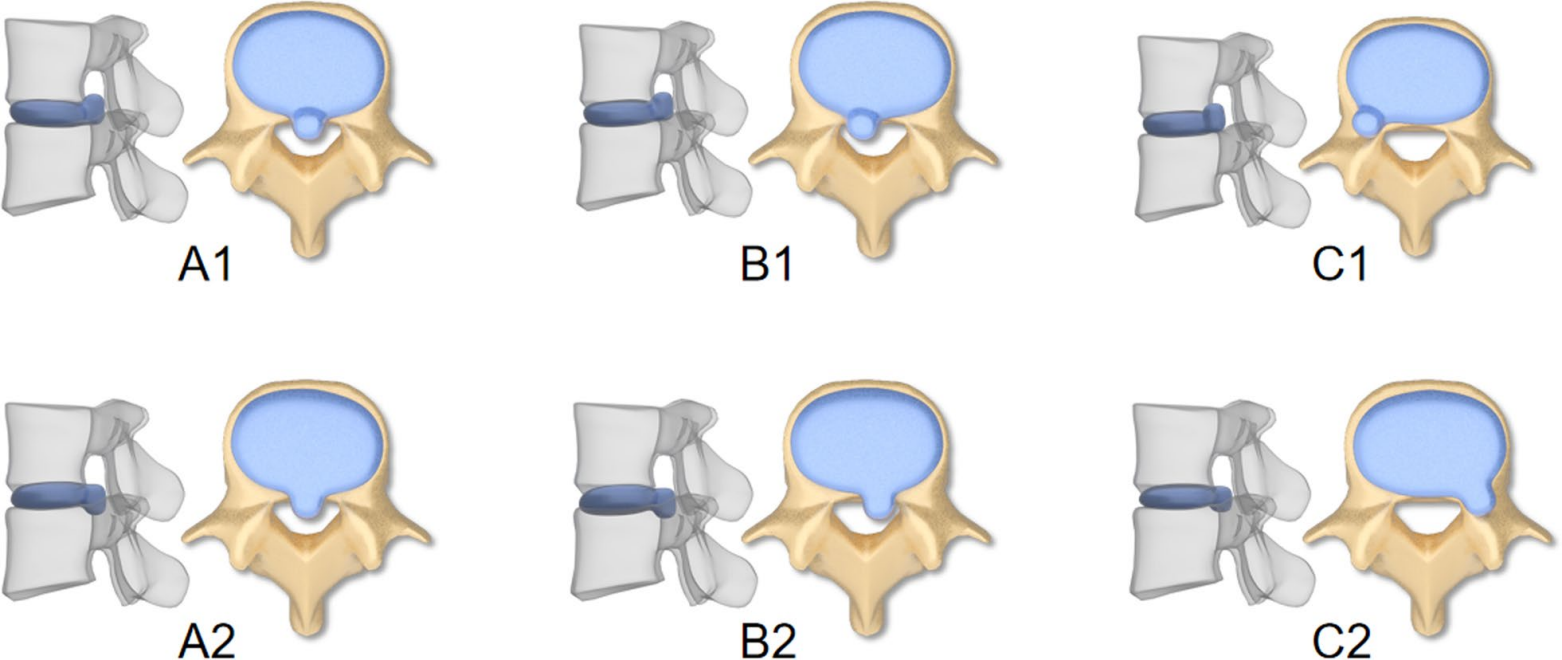
Schematic diagram of the modified classification

**Table 2:** Patient characteristics

Variable	Migrated (*n* = 105)	Non-migrated (*n* = 895)	*P*
Gender (*n*)			
Female	50	423	0.835
Male	55	472	
Age (yr)	52.30 ± 14.37	51.62 ± 13.38	0.683
Level			
L2–3	1	10	0.756
L3–4	9	56	
L4–5	50	428	
L5–S1	45	401	
Course(n)			
n ≤ 3 months	47	318	0.686
3 months < *n* < 1 year	10	142	
n ≥ 1 year	48	435	
Chronic disease (*n*)			
Hypertension	47	405	0.442
Diabetes	40	385	0.581
BMI	23.10 ± 2.37	21.08 ± 2.16	0.042

## Material and methods

### Patient selection

After the approval of the Ethics Committee of Hangzhou Hospital of Traditional Chinese Medicine (approval number: 2022KY022), we applied the modified classification system in patients with MLDH treated in our hospital from January 2016 to December 2019. This study was a retrospective study and informed consent was not required. Inclusion criteria: (1) MLDH was confirmed by imaging examination (MRI and CT); The symptoms, signs and imaging were consistent, and all were a single responsible segment. (2) low back pain with or without lower limb pain, numbness or intermittent claudication, and Oswestry Disability Index (ODI) > 30%. Exclusion criteria: (1) Combined with lumbar spondylolisthesis and spinal instability; (2) Significant scoliosis, kyphosis or structural sagittal imbalance.

### Reliability for modified classification

We identified three subjects with different experiences (residents, < 5 years of work; Attending physician, working time 5–10 years; Associate chief physician, working time > 10 years). The modified classification of 105 cases of migrated lumbar spine MRI was performed twice. All the patients underwent complete MRI reconstruction scanning in our hospital using the same equipment, and all the image data did not contain any information and markers related to classification. Firstly, three doctors were trained on improved classification and distributed training materials (including classification and typical image legends). After the training, the imaging data of the patients were randomly numbered, and the modified classification of migrated nucleus pulposus was judged by three doctors independently. Two weeks later, the sorting data of the patients were shuffled and the classification was judged again by three doctors. The double blind principle was adopted for the two ranking and judgment. The Cohens' kappa or Fleiss' Kappa coefficient was used to evaluate the consistency and reproducibility of the classification. Typing evaluation included both the major subtypes (type A, B, and C) and each subtype (A1, A2, B1, B2, C1, C2).

### Clinical application for modified classification

The included patients were followed up for 2 years. During the follow-up period, the treatment plan, operation time, surgical approach, VAS score, ODI score and other relevant information were collected to evaluate the clinical effectiveness of the modified classification. The general data of patients such as gender, age, responsible segment, course of disease and underlying diseases were collected. Logistic regression and ROC analysis were used to analyze the risk factors of surgical treatment in patients with MLDH.

### Statistical analysis

SPSS 26.0 software was used to analyze the data. Kappa coefficient (K) was calculated to evaluate the reliability (inter-observer consistency) and the repeatability (intra-observer repeatability) of the two classifications by the same observer. The larger the *K* value, the higher the reliability. The *K* value ≤ 0.40 indicates that it is not reliable, 0.41–0.60 indicates that it is generally reliable, 0.61–0.80 indicates that it is basically reliable, and 0.81–1.00 indicates that it is highly reliable. Normally distributed data sets are presented as mean ± standard deviation. Data sets that were not normally distributed were presented as medians, and statistical significance was assessed using the Wilcoxon test or Kruskal–Wallis test. *P* < 0.05 were considered to indicate a significant difference.

## Results

### Demographics for patients

A total of 1000 lumbar MRI were retrieved in this study, including 527 males and 473 females. There were 452 patients with hypertension and 425 patients with diabetes. The average age was 52.08 ± 14.15 (18–88) years, and the average BMI was 22.65 ± 2.46. We found a statistically significant difference in BMI between the two groups.

### Reliability for modified classification

In the two classification processes, a total of 630 classification evaluations were performed by three spine surgeons. The most common type is type B2, and the least common type is type C1.

#### Intraobserver repeatability analysis

The intraobserver average Kappa coefficient of the modified migrated nucleus pulposus classification was 0.839, which had a high degree of repeatability. Type A and type B had excellent repeatability, and the average Kappa coefficients were 0.886 and 0.828, respectively. The repeatability of type C was relatively poor, and the average Kappa coefficient was 0.804. The reproducibility of each subtype was further tested, and the reproducibility of each subtype was medium to high (Table 3, 4).

**Table 3:** Reliability for the main types of intraobserver

Type	Observer 1	Observer 2	Observer 3
	K	K	K
A	0.851	0.894	0.913
B	0.829	0.790	0.866
C	0.866	0.811	0.735

**Table 4:** Reliability analysis of the subtypes of intraobserver

Type	Observer 1	Observer 2	Observer 3
	K	K	K
A1	0.947	1	1
A2	0.898	0.871	0.892
B1	0.886	0.962	0.922
B2	0.872	0.772	0.858
C1	0.904	0.904	1
C2	0.864	0.779	0.630

#### Interobserver consistency analysis

The average interobserver Kappa coefficient of the modified migrated nucleus pulposus classification was 0.828, which had high consistency. The determination of type A and type B had medium to high consistency and excellent repeatability, and the average Kappa coefficients were 0.886 and 0.822, respectively. The consistency of type C was relatively poor, and the average Kappa coefficient was 0.776. The consistency of each subtype was further tested, and the consistency of A1 type was the best, with an average Kappa coefficient of 0.947, and the consistency of C2 type was the worst, with an average Kappa coefficient of only 0.689 (Table 5, 6).

**Table 5:** Reliability for the main types of interobserver

Type	The first judgment	Second judgment
	K (95% CI)	K (95% CI)
A	0.800 (0.796–0.804)	0.971 (0.968–0.975)
B	0.695 (0.692–0.699)	0.949 (0.946–0.953)
C	0.602 (0.599–0.606)	0.950 (0.946–0.954)

**Table 6:** Reliability analysis of the subtypes of interobserver

Type	The first judgment	Second judgment
	K (95% CI)	K (95% CI)
A1	0.930 (0.927–0.934)	0.964 (0.961–0.968)
A2	0.790 (0.786–0.794)	0.948 (0.944–0.952)
B1	0.875 (0.871–0.878)	0.975 (0.971–0.979)
B2	0.708 (0.704–0.711)	0.959 (0.955–0.962)
C1	0.802 (0.799–0.806)	0.934 (0.931–0.938)
C2	0.486 (0.482–0.489)	0.893 (0.889–0.896)

### Clinical application for modified classification

#### Characteristics of patients

**Fig. 2:**
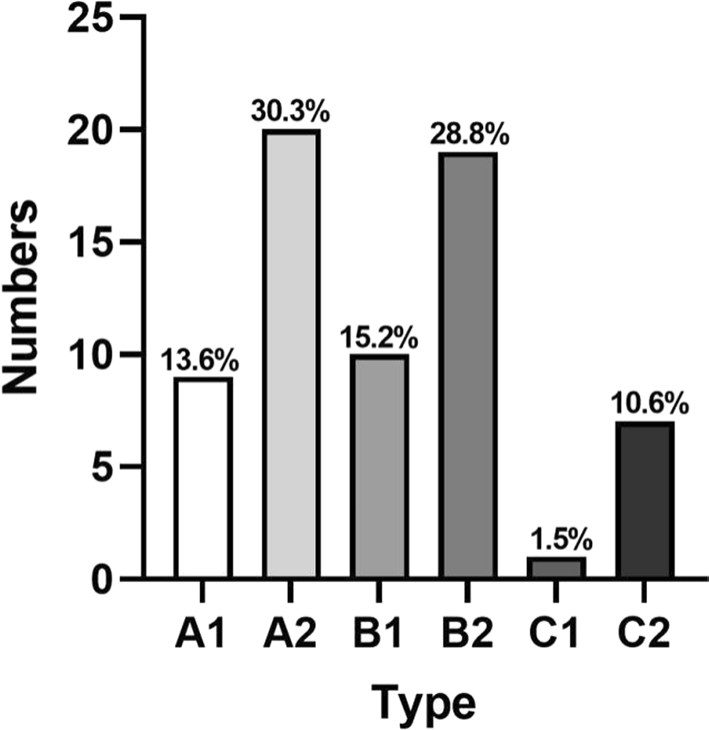
Modified classification of conservative patients

**Fig. 3:**
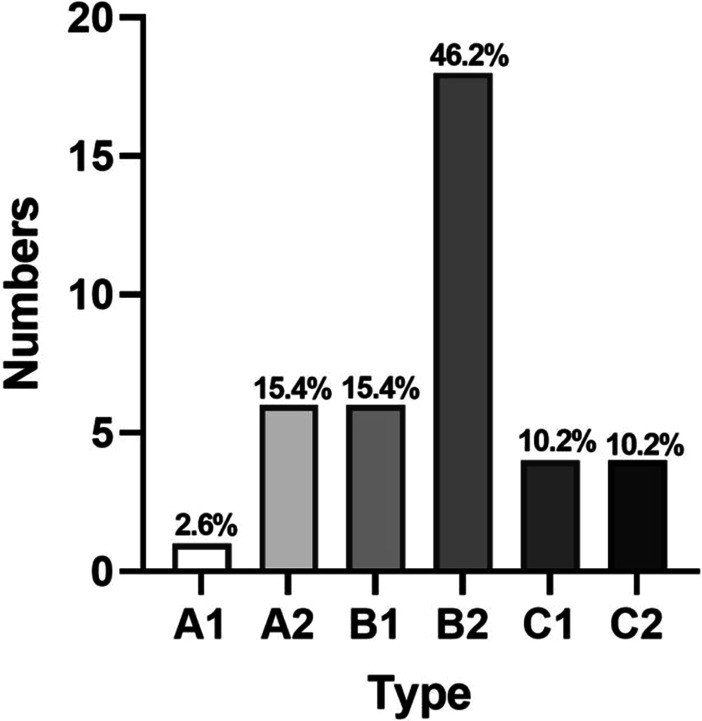
Modified classification of surgical patients

Table 7 shows the detailed baseline characteristics of all patients. A total of 105 patients were enrolled in this study, including 55 males and 50 females. There were 47 patients with hypertension and 40 patients with diabetes. The average age was 52.30 ± 14.37 (18–83) years, and the average BMI was 23.1 ± 2.37. All patients were followed up for 2 years. During the follow-up period, 66 patients (62.9%) received conservative treatment and 39 patients (37.1%) underwent surgical treatment. Figures 2 and 3 show the modified classification characteristics of the patients.

**Table 7:** Patient characteristics (conservative vs. surgical)

Variable	Conservative (*n* = 66)	Surgical (*n* = 39)	*P*	*X*^2^	*t*
Gender (*n*)					
Female	26	24	0.959	0.003	
Male	40	15			
Age (yr)	52.88 ± 14.10	51.33 ± 15.02	0.597		0.531
Level					
L2–3	1	0			
L3–4	5	4	0.418	0.655	
L4–5	35	15	0.876	0.024	
L5–S1	25	20	0.770	0.086	
Course (*n*)					
* n* ≤ 3 months	36	11	0.004		− 2.988
3 months < *n* < 1 year	7	3			
*n* ≥ 1 year	23	25			
Chronic disease (*n*)					
Hypertension	30	17	0.621	0.244	
Diabetes	25	15	0.918	0.011	
BMI	23.00 ± 2.24	23.27 ± 2.61	0.241		− 1.180

#### Clinical effectiveness of modified classification

**Fig. 4:**
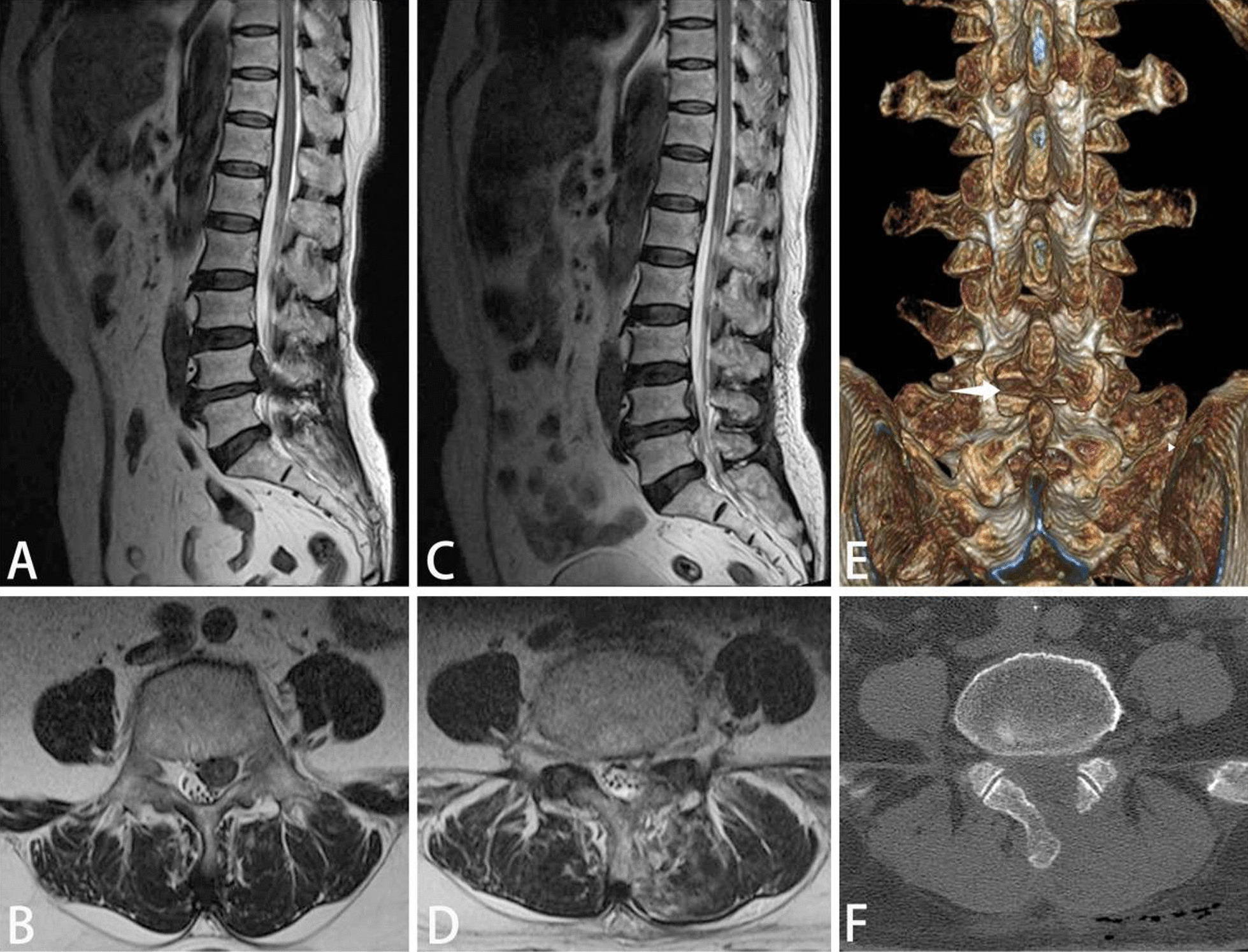
A 65-year-old female patient was diagnosed as type B1 according to the modified classification. The nucleus pulposus was removed through the posterior approach. **A**–**B**: The migrated nucleus pulposus was seen on preoperative MRI. **C**–**D**: Postoperative MRI showed that the migrated nucleus pulposus was completely removed. **E**: The bony tunnel of the lamina was seen in the postoperative CT three-dimensional image. **F**: postoperative vertebral CT showed partial bone defect in the left lamina

**Fig. 5:**
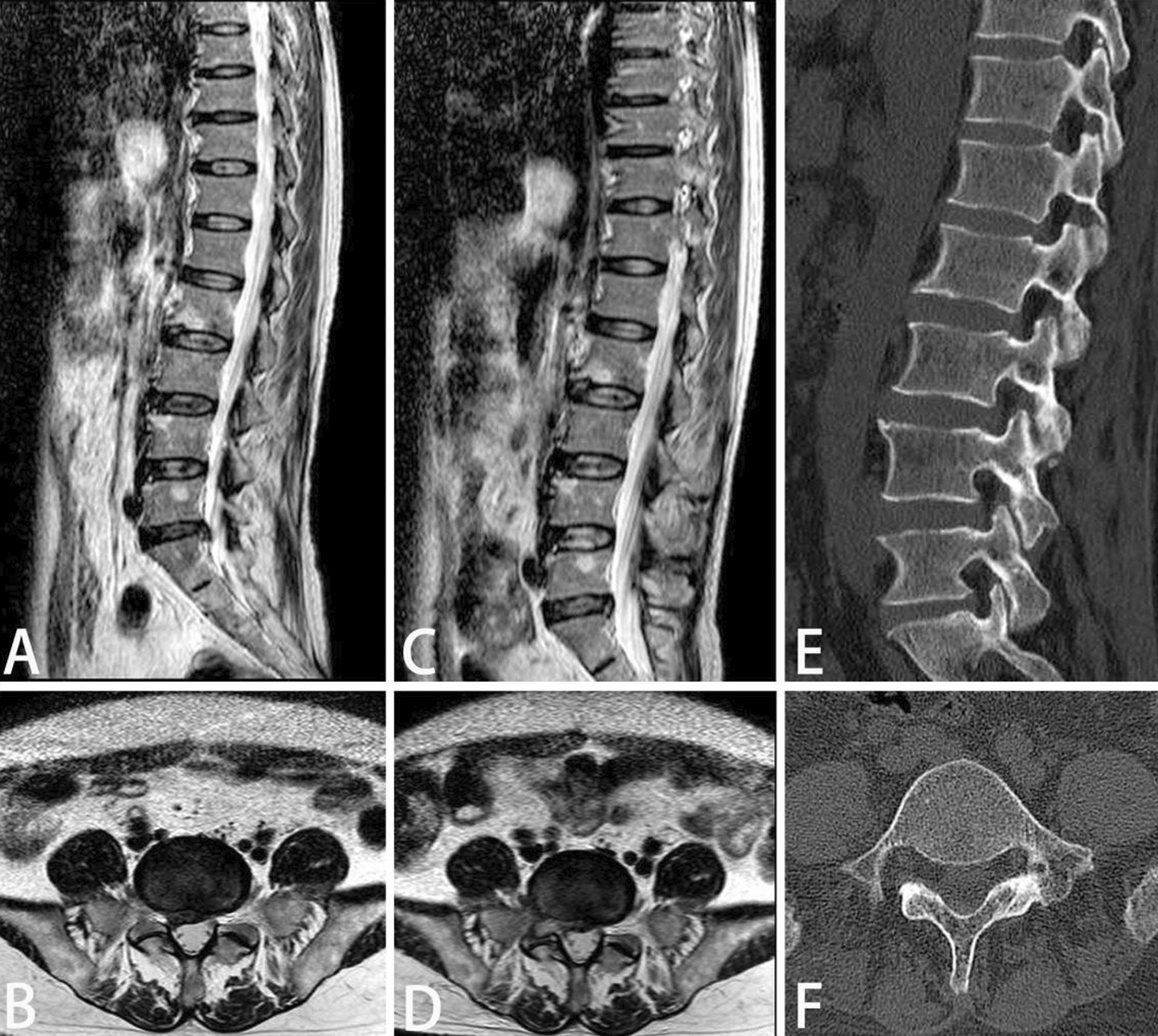
A 64-year-old male patient was diagnosed as type B2 by modified classification. The nucleus pulposus was removed through lateral approach. **A**–**B**: Right prolapsed nucleus pulposus was seen on preoperative MRI. **C**–**D**: Postoperative MRI showed that the nucleus pulposus was completely removed. **E**–**F**: postoperative vertebral CT showed partial bone defects in the foraminal area

In this study, 39 patients were treated with surgery, and the rate was 37.1%. All surgical patients were treated with percutaneous transforaminal endoscopic discectomy. Among 39 patients, 10 cases were operated via posterior approach and 29 cases were operated via lateral approach. The migrated nucleus pulposus was completely removed in all patients, and no recurrence or secondary fracture occurred during the follow-up period. The low back pain and ODI index were significantly improved in all patients at 1 year after operation (Table 8). Typical cases are shown in Figs. 4 and 5.

**Table 8:** Comparison of VAS and ODI during perioperative period

Time	Lateral approach (*n* = 29)		Posterior approach (*n* = 10)	
	VAS	ODI (%)	VAS	ODI (%)
Preoperative	5.92 ± 0.96	41.26 ± 4.97	5.86 ± 1.04	44.61 ± 4.82
Immediate postoperative	3.13 ± 0.73		3.00 ± 0.77	
1 year after surgery	1.28 ± 0.46	22.36 ± 3.71	1.29 ± 0.46	22.11 ± 3.71
P	< 0.05	< 0.05	< 0.05	< 0.05

#### Analysis of surgical risk factors in patients with Migrated Lumbar Disc Herniation

**Fig. 6:**
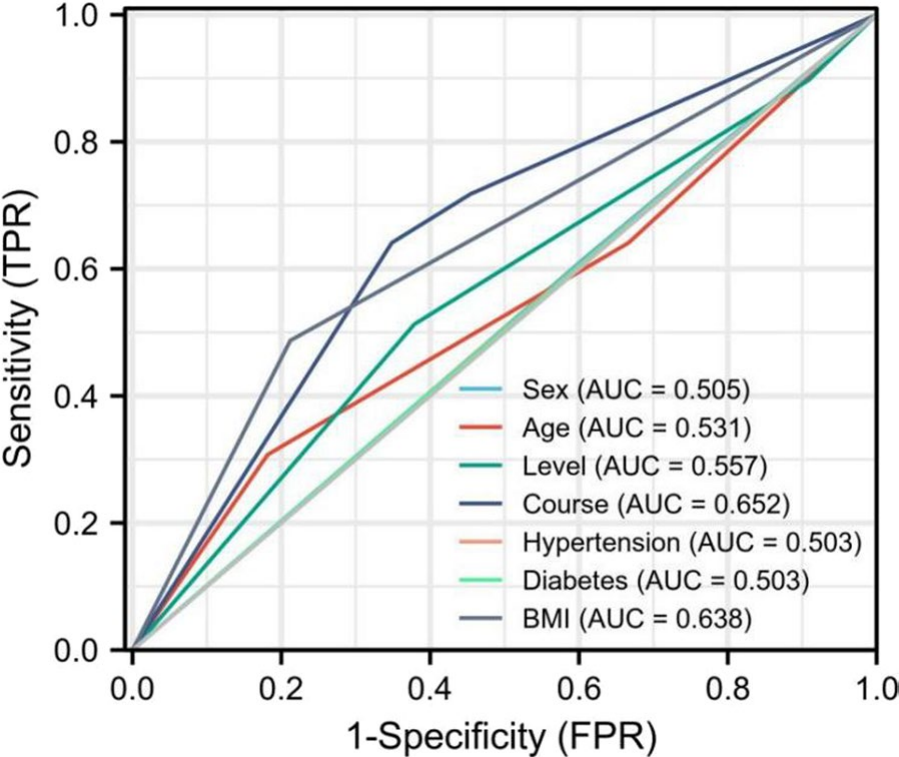
ROC analysis showed that the course of disease (≥ 1 year) and BMI (≥ 24) were independent risk factors for surgical treatment with MLDH

We collected the data of gender, age, level, course of disease, BMI, and underlying diseases of the included patients, and analyzed the risk factors with whether the patient underwent surgery during the follow-up period as the outcome index. Logistic regression (Table 9) and ROC analysis (Fig. 6) showed that the course of disease (≥ 1 year) and BMI (≥ 24) were independent risk factors for surgical treatment in patients with MLDH.

**Table 9:** Logistic regression analysis of risk factors for surgical treatment

	OR (95% CI)	*P*
Univariate analysis		
Gender	0.962 (0.427–2.167)	0.925
Age	0.836 (0.492–1.420)	0.507
Level	1.352 (0.721–2.538)	0.347
Course	1.896 (1.218–2.951)	0.005
BMI	3.561 (1.499–8.458)	0.004
Hypertension	1.032 (0.450–2.367)	0.941
Diabetes	1.122 (0.478–2.633)	0.792
Multivariate analysis		
Course	0.548 (0.346–0.868)	0.010
BMI	0.309 (0.127–0.753)	0.010
Constant	0.000	0.000

## Discussion

MLDH is common in clinical practice, which can occur at any age [12]. It is often accompanied by severe pain that is not relieved by positioning, bed rest, or the use of analgesics [13]. The main reason is that the migrated nucleus pulposus tissue not only causes physical compression to the nerve, but also causes sterile inflammatory reaction [4–6]. The incidence of cauda equina injury is significantly higher than that of other prominent types, which is extremely harmful to society [14, 15]. It has been reported that the clinical incidence of MLDH is 1.7–3% [2, 3]. Our data suggest that the prevalence of MLDH can reach a staggering 10.5%. Through the analysis of the general data of the patients, we found that the average BMI of patients with migrated nucleus pulposus was higher than that of patients without migrated nucleus pulposus (*P* < 0.05), which suggested that our obese patients may be more likely to develop MLDH.

An ideal classification system can not only help clinicians diagnose and determine treatment plans, but also guide the operation process [16]. Lumbar MRI can clearly show the specific location and shape of the migrated nucleus pulposus, and the images obtained are more complete and comprehensive [8]. At present, there are two types of migrated nucleus pulposus classification recognized by most scholars based on lumbar MRI. One is that Hu et al. [10] divided the protruding nucleus pulposus into four zones at the transverse position by dividing the posterior edge of the vertebral body into zones 1, 2, 3, and 4. 1 and 2 are the internal border of the pedicle on both sides, that is, the anterior border of the spinal canal, which is divided into three equal parts. The middle 1/3 was zone 1, and the left and right 1/3 were zone 2. Area 1 is called the central area; Area 2 is called paracentral area; Zone 3 was called lateral zone, which was between the inner and outer parts of the pedicle, that is, between the foramen boundary; Zone 4 is called the extreme lateral zone and is outside the lateral aspect of the pedicle. The paracentral area, lateral area and extreme lateral area were divided into left and right sides. According to the sagittal MRI images, Lee et al.^[11]^ divided the migrated nucleus pulposus into four zones: zone I was 3 mm above the midline of the pedicle of the upper vertebral body; Zone II was 3 mm below the midline of the pedicle of the upper vertebral body to the lower edge of the upper vertebral body; Zone III was the midline between the upper edge of the lower vertebral body and the lower pedicle. Zone IV is distal to the midline of the inferior pedicle. However, in clinical practice, neither of the two types used alone can show the position of the migrated nucleus pulposus stereoscopically, which lacks guiding significance for the planning of the puncture Angle before percutaneous transforaminal endoscopic discectomy. Based on the above two types, our team proposed A simplified fusion classification system for the migrated nucleus pulposus, which retained the original method of transection and renamed it as the three main types A,B, and C. At the same time, the upward or downward detachment of the nucleus pulposus was used as the criterion for the classification system.

Reliability evaluation is a scientific and practical method to evaluate a new classification, which is widely used in clinical practice [17]. In this study, we found that both the intra-and inter-observer mean Kappa coefficient (K) was greater than 0.8. We confirmed that the modified nucleus pulposus classification system had generally good inter-and intra-observer agreement. We found that type C2 had the worst agreement, which may be due to the difficulty of diagnosis of migrated nucleus pulposus in the lateral foramen area, which is easily judged as type B2. Type A1 has the best consistency, indicating that this classification diagnosis is easier. This is the first evaluation of the consistency and reproducibility of this classification by our team after the proposal of this classification. We included data from 105 cases, and the classification was evaluated by multiple observers with different clinical experiences, which makes the results more reliable.

Our results show that the modified classification system has good clinical application value. On the one hand, we found that types A2 and B2 accounted for 59.1% of the patients treated conservatively. Therefore, for most patients with MLDH, especially those with A2 and B2 types, Conservative treatment can be attempted. On the other hand, the modified classification system can restore the three-dimensional position of the migrated nucleus pulposus in the spinal canal, which has a good guiding significance for preoperative puncture target estimation, puncture Angle planning and decompression range determination. In this study, the migrated nucleus pulposus was completely removed in 39 patients by lateral approach or posterior approach under the guidance of the modified classification. In addition, the results of logistic regression and ROC analysis show that ≥ 1 year and BMI ≥ 24 may be independent risk factors for surgical treatment of patients with lumbar disc herniation. Spine surgeons should be on high alert for these two types of MLDH patients, who have potential surgical risks.

Percutaneous transforaminal endoscopic discectomy has the advantages of minimal trauma, rapid recovery, low incidence of complications, exact curative effect, and maximum retention of spinal stability [18, 19]. It has become the main method for the treatment of lumbar disc herniation, with an effective rate of 88–92% [20]. However, the removal of migrated nucleus pulposus through a single endoscopic approach is limited for the treatment of the intervertebral space, which is easy to cause residual nucleus pulposus at the level of the intervertebral space, reherniation of nucleus pulposus, and recurrence of disc herniation [21–23].Our study show the modified classification was used to roughly predict the spatial position of the migrated nucleus pulposus before operation, select the appropriate lateral or posterior approach, and use different bone channels to perform target puncture according to the main position of the migrated nucleus pulposus, so as to reserve the visual field and operation space under endoscope for the intervertebral space treatment, and reduce the possibility of herniation recurrence as much as possible. For the upward mobilization type, because the lateral approach is easy to irritate the nerve root [24], we suggest that the A1 and B1 type can be removed through the natural space of the upper and lower laminae. For type C1, because the nucleus pulposus reaches the foraminal area, the dorsal approach requires a large amount of traction of the nerve root, which will cause nerve root injury, and can be removed directly through the lateral transforaminal approach. For type A2, B2 and C2, the downward migrated nucleus pulposus can also be removed directly through the lateral approach.

This study also has some limitations; first, we selected cases from the imaging database and decided which cases were analyzed and compared, which may have some selection bias. In addition, the observer's training level and work experience may also affect the results of classification. This study is a retrospective study with limited sample size and relatively short follow-up time. Further prospective and randomized trials are needed to verify the guiding value of this classification system in clinical practice.

## Conclusion

Our study found that there was a high incidence of migrated nucleus pulposus in patients with lumbar disc herniation, and patients with higher BMI were more likely to have this disease. Based on MRI of the lumbar spine, we proposed a modified classification system of migrated nucleus pulposus for the first time, and confirmed that this classification system has good consistency and repeatability. On the basis of ignoring the primary segment, we believe that conservative treatment is the first choice for most patients with MLDH, especially for type A2 and B2 patients. Patients with disease duration ≥ 1 year and BMI ≥ 24 have potential surgical risk. For patients with type A1 and B1 according to the modified classification, the posterior approach can be used for treatment, while the lateral approach is recommended for patients with type A2, B2, C1 and C2.
